# Constructing Soft Perovskite–Substrate Interfaces for Dynamic Modulation of Perovskite Film in Inverted Solar Cells with Over 6200 Hours Photostability

**DOI:** 10.1002/advs.202202028

**Published:** 2022-08-17

**Authors:** Wenxuan Lv, Zhaoying Hu, Wei Qiu, Dongdong Yan, Meicheng Li, Anyi Mei, Ligang Xu, Runfeng Chen

**Affiliations:** ^1^ Key Laboratory for Organic Electronics and Information Displays (KLOEID) & Jiangsu Key Laboratory for Biosensors Institute of Advanced Materials (IAM) Nanjing University of Posts & Telecommunications 9 Wenyuan Road Nanjing 210023 China; ^2^ State Key Laboratory of Alternate Electrical Power System with Renewable Energy Sources School of New Energy North China Electric Power University Beijing 100192 China; ^3^ Wuhan National Laboratory for Optoelectronics Huazhong University of Science and Technology Wuhan Hubei 430074 China

**Keywords:** device stability, dynamic modulation, passivation, perovskite solar cells, residual strain

## Abstract

High‐performance perovskite solar cells (PSCs) depend heavily on the quality of perovskite films, which is closely related to the lattice distortion, perovskite crystallization, and interfacial defects when being spin‐coated and annealed on the substrate surface. Here, a dynamic strategy to modulate the perovskite film formation by using a soft perovskite–substrate interface constructed by employing amphiphilic soft molecules (ASMs) with long alkyl chains and Lewis base groups is proposed. The hydrophobic alkyl chains of ASMs interacted with poly(triarylamine) (PTAA) greatly improve the wettability of PTAA to facilitate the nucleation and growth of perovskite crystals, while the Lewis base groups bound to perovskite lattices significantly passivate the defects in situ. More importantly, this soft perovskite–substrate interface with ASMs between PTAA and perovskite film can dynamically match the lattice distortion with reduced interfacial residual strain upon perovskite crystallization and thermal annealing owing to the soft self‐adaptive long‐chains, leading to high‐quality perovskite films. Thus, the inverted PSCs show a power conversion efficiency approaching 20% with good reproducibility and negligible hysteresis. More impressively, the unencapsulated device exhibits state‐of‐the‐art photostability, retaining 84% of its initial efficiency under continuous simulated 1‐sun illumination for more than 6200 h at elevated temperature (≈65 °C).

## Graphical abstract

A dynamic modulation strategy is developed to fabricate high‐quality perovskite films by constructing soft perovskite‐substrate interfaces. As a result, the unencapsulated device shows state‐of‐the‐art photostability, maintaining 84% initial efficiency under continuous simulated 1‐sun illumination for more than 6200 h.

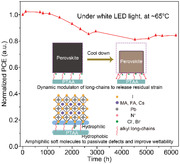

## 1 Introduction

Perovskite solar cells (PSCs) have made breathtaking development over the past decade owing to their excellent optoelectronic properties, such as high carrier mobility, long carrier diffusion length, tunable bandgaps, elevated absorption coefficient, low exciton binding energy, and relevant solution processability.^[^ ^1^, ^2^, ^3^, ^4^, ^5^, ^6^ ^]^ The state‐of‐the‐art PSCs often show T_80_ lifetime (the time required to lose 20% of the initial efficiency) values above 1000 h under illumination at 60–70 °C.^[^ ^7^, ^8^, ^9^, ^10^ ^]^ However, the lifespan of PSCs still lags far behind that of commercial silicon cells with less than 0.5% annual degradation rate at elevated temperatures (60–85 °C).^[^ ^11^, ^12^, ^13^ ^]^ To stabilize solar devices, various encapsulating techniques have been used to protect against moisture and oxygen.^[^ ^14^, ^15^, ^16^ ^]^ Unfortunately, the intrinsic instability caused by the perovskite film (ion migration, phase separation, and defects, etc.) during operational conditions cannot be overcome by encapsulation methods.^[^ ^17^, ^18^ ^]^ Thus, the most important prerequisite for improved intrinsic stability of PSCs is regulating the quality of perovskite films. Various attempts have been made to achieve high‐quality perovskite films for improving the performance of PSCs, including vacuum or thermal assisting,^[^ ^19^ ^]^ vapor incubation,^[^ ^20^ ^]^ additive engineering,^[^ ^21^ ^]^ post‐treatment and passivation,^[^ ^22^ ^]^ and liquid medium annealing.^[^ ^23^ ^]^

The perovskite–substrate interfaces strongly affect perovskite crystallization, interface defects, and residual strain, which play a key role in fabricating high‐quality perovskite films. In general, poly(triarylamine) (PTAA) used as the hole‐transporting layer (HTL) for perovskite–substrate interface is highly efficient for inverted PSCs despite the unsatisfactory perovskite growth by hydrophobic PTAA to generate rough perovskite films with numerous pinholes.^[^ ^24^, ^25^, ^26^ ^]^ Meanwhile, abundant defects, such as ion vacancies, substitutions, and interstitial/undercoordinated species exist at perovskite–substrate interfaces, which are conducive to ion migration, non‐radiative charge recombination, hysteresis, and instability of devices.^[^ ^27^, ^28^ ^]^ In addition, the mismatch in thermal expansion coefficients between the perovskite (≈3.3–8.4 × 10^−5^ K^−1^) and substrate (≈0.4–1.0 × 10^−5^ K^−1^) generate residual strain at the perovskite‐substrate during cooling of annealed perovskite films to room temperature, leading to lattice distortion and acceleration of perovskite degradation.^[^ ^29^, ^30^ ^]^ Therefore, improving the wettability of PTAA, passivating the perovskite–substrate interfaces defects, and releasing residual strain to achieve high‐quality perovskite films are highly desirable features for high‐performance inverted PSCs.

In this study, soft perovskite–substrate interfaces are constructed for dynamic modulation of perovskite film formation to improve the intrinsic stability of PSCs by employing amphiphilic soft molecules (ASMs), such as cetyltrimethylammonium bromide (CTAB), dodecyltrimethylammonium bromide (DTAB), and dodecyltrimethylammonium chloride (DTAC). This soft perovskite–substrate interfaces can dynamically match the lattice distortion with reduced interfacial residual strain upon perovskite crystallization and thermal annealing owing to the soft self‐adaptive long‐chains. Moreover, such ASMs could improve the wettability of PTAA and passivate the perovskite–substrate defects to suppress non‐radiative charge recombination. As a result, the unencapsulated device remained over 84% of initial efficiency under continuous 1‐sun illumination at ≈65 °C for more than 6200 h, which is the best photostability achieved in inverted PSCs to date. Moreover, the optimized photovoltaic device showed a power conversion efficiency (PCE) approaching 20% with good reproducibility and negligible hysteresis. Overall, this dynamic strategy based on ASMs provides important clues in the modulation of perovskite crystallization, highlighting the great potential of soft perovskite–substrate interfaces in constructing high‐quality perovskite films for highly stable and efficient photovoltaic devices.

## 2 Results and Discussion

**Figure 1:**
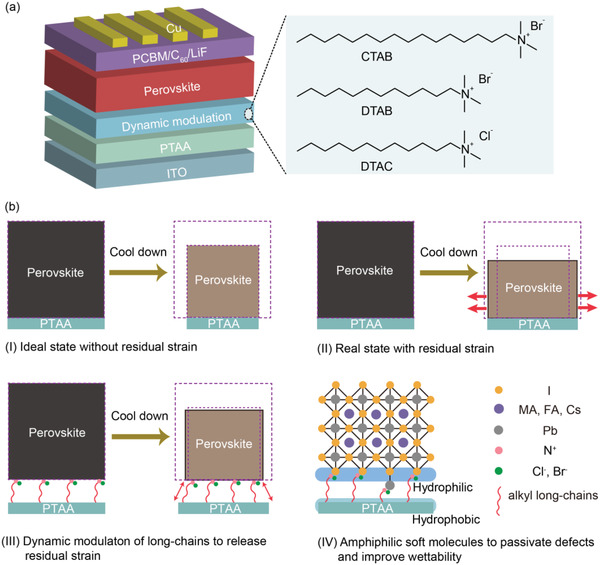
a) Device structure and chemical structures of CTAB, DTAB, and DTAC. b) Schematic illustration of I) ideal volume contraction of the annealing perovskite films after cooling down, II) residual strain due to substrate adhesion for PTAA‐based perovskite film, III) residual strain release for ASMs‐based perovskite film, IV) schematic illustration of passivated perovskite‐substrate defects and improved wettability of perovskite precursor on PTAA by a multifunctional ASMs.

The device structure of inverted PSCs is schematically represented in **Figure** **1**a. As can be seen, ASMs (CTAB, DTAB, and DTAC) were deposited on the top surface of PTAA by spin‐coating. Perovskite films based on (Cs_0.05_FA_0.81_MA_0.14_)Pb(I_0.86_Br_0.14_)_3_ were then fabricated by spin‐coating on the ASMs. It should be noted that ASMs could not form self‐assembled monolayer due to no existing intense interaction forces between ASMs and PTAA. The ASMs with good wettability simultaneously improved the wettability of perovskite precursor on PTAA since the molecules consisted of hydrophilic Lewis base groups and hydrophobic long alkyl chain, features suitable for passivating the perovskite–substrate defects and reducing residual strain to dynamically modulate the quality of perovskite film for high‐performance inverted PSCs (Figure 1b).

**Figure 2:**
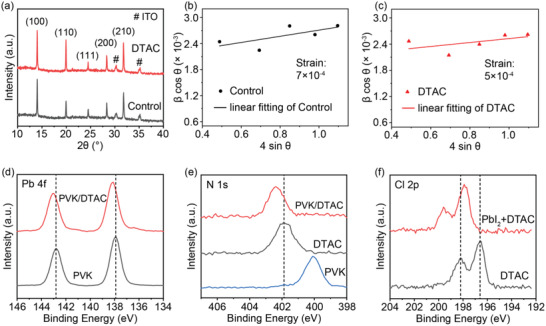
a) XRD patterns of perovskite films deposited on PTAA and DTAC. Williamson–Hall plot of full width at half maximum (FWHM) corresponding to the XRD peaks. The lattice strains of perovskite films deposited on b) PTAA and c) DTAC were calculated as the slope of the linear fitting. XPS spectra of d) Pb 4f for pure perovskite (PVK) and DTAC on perovskite film, e) N 1s for pure DTAC and DTAC on perovskite film, and f) Cl 2p for pure DTAC and DTAC doping with PbI_2_ (1:1 m/m).

To investigate the effects of dynamic modulation strategy on residual lattice strain (*ε*) and perovskite crystallization, X‐ray diffraction (XRD) of annealed perovskite films deposited on PTAA and DTAC after cooling were recorded. As shown in **Figure** **2**a, the peak intensity of (100) orientation for DTAC‐derived perovskite film was 104% of that for the control perovskite films, demonstrating that the perovskite crystals preferentially grow on DTAC and significantly improve crystallinity.^[^ ^31^ ^]^ In addition, DTAC‐derived perovskite film displayed the lowest full width at half maximum of (100) plane. Thus, DTAC could induce a better crystallization with larger crystallite sizes. The Williamson–Hall equation was adopted for estimating the *ε* using the above XRD results through the slope of the fitting line (a detailed description can be found in the Supporting Information). As presented in Figure 2b,c, the *ε* of DTAC‐derived perovskite film decreased to 5 × 10^–4^ when compared to the control with 7 × 10^–4^, suggesting the release of a residual strain of perovskite–substrate interfaces by the dynamic modulation of soft self‐adaptive long‐chains.^[^ ^32^ ^]^

Based on the above data, a mechanism of residual strain was proposed for perovskite films. As shown in Figure 1b, the volume contraction of the perovskite film for the ideal state without residual strain (Figure 1b(I)) was consistent with that of the substrate from thermal annealing to room temperature. However, residual strain emerged owing to the substrate adhesion limiting the contraction of perovskite film issued from its larger thermal expansion coefficient when compared to those of PTAA film and indium tin oxide substrate shown in Figure 1b(II). For perovskite film with DTAC, the residual strain was reduced by the dynamic modulation strategy due to the soft self‐adaptive long‐chains with compression or stretching, which could help decline the adhesion of the PTAA substrate and self‐adapt the contraction mismatch between perovskite and substrate (Figure 1b(III)).

X‐ray photoelectron spectroscopy (XPS) was performed to investigate the existence of chemical interactions between DTAC and perovskite films. As shown in Figure 2d–f, the XPS patterns of Pb 4f for perovskite/DTAC displayed two peaks at 138.1 and 143.0 eV; compared to pure perovskite film with two peaks located at 137.9 and 142.8 eV, the binding energy of Pb 4f shifted by 0.2 eV toward higher values, indicating clear chemical interactions between DTAC and Pb atom of perovskite (Figure 2d). In addition, the N 1s changed from 401.9 to 402.4 eV from DTAC to DTAC on perovskite films. The Cl 2p shifted by 1.3 eV from DTAC to PbI_2_ mixed DTAC films (Figure 2e,f), further demonstrating the heavy chemical interactions between Lewis base groups in DTAC and Pb in perovskite at the interface. Such interactions could passivate the undercoordinated Pb^2+^ defects and suppress ion migration.^[^ ^5^, ^33^, ^34^, ^35^, ^36^ ^]^ Moreover, lattice mismatch could be reduced due to heavy chemical interaction between perovskite film and ASM.

**Figure 3:**
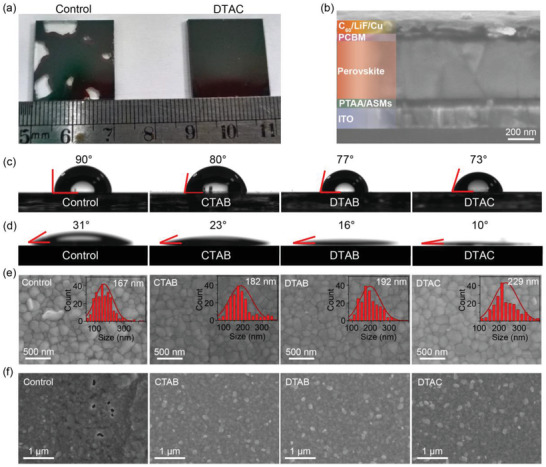
a) Digital photographs of perovskite films deposited on pure PTAA (left) and DTAC (right). b) Cross‐sectional SEM image of the inverted device based on ASMs. Contact angles of c) water and d) perovskite precursor on PTAA and ASMs surfaces. SEM images of thick and thin perovskite films based on PTAA and ASMs cast from e) 1.4 and f) 0.3 mol L^−1^.

To investigate whether the deposition of ASMs improved the wettability of PTAA surface, the perovskite precursor was spin‐coated on the top of PTAA or ASMs. Photographs of the spin‐coated perovskite films (20 mm × 20 mm) are provided in **Figure** **3**a. Compared to the perovskite formed on PTAA showing incomplete surface coverage, the perovskite layer based on DTAC depicted complete surface coverage with homogeneously formed film. The cross‐sectional scanning electron microscopy (SEM) image of perovskite film deposited on DTAC displayed suppressed pinholes and interspace when compared to perovskite film deposited on PTAA (Figure 3b and Figure S1, Supporting Information). Also, the cross‐sectional SEM image confirmed the formation of ≈60 nm thick PTAA and ASMs. The perovskite layer revealed a thickness of ≈400 nm, and a ≈100 nm thick Cu electrode was evaporated on the top of C_60_/LiF layers.

To quantitatively investigate the surface wettability of PTAA or ASMs, contact angle measurements were further conducted and the results were gathered in Figure 3c,d. The water contact angle of PTAA was estimated to be 90°, while low values of 80°, 77°, and 73° were recorded for PTAA/CTAB, PTAA/DTAB, and PTAA/DTAC, respectively. Hence, the hydrophilicity of PTAA was improved by the presence of ASMs (Figure 3c). In this case, the HTL became more affiliated with the polar perovskite solution for facilitating the nucleation and growth of perovskite crystals. In addition, the contact angles of perovskite precursors illustrated in Figure 3d further proved ASMs more affiliated to the perovskite precursor than PTAA, favorable for fabricating high‐quality perovskite films.

The crystal growth of perovskite on PTAA and ASMs was explored by SEM characterization. As shown in Figure 3e,f, thick (≈400 nm) and thin (≈85 nm) perovskite films, representing the bulk and interfacial morphologies of films, were successfully fabricated by spinning 1.4 and 0.3 mol L^−1^ perovskite precursor solutions on the top of PTAA and ASMs surfaces, respectively.^[^ ^24^ ^]^ The results demonstrated a better crystal growth in all thick perovskite films deposited on CTAB, DTAB, and DTAC. The average grain sizes rose from 167 nm for perovskite on PTAA to 182, 192, and 229 nm for perovskite on CTAB, DTAB, and DTAC, respectively (Figure 3e). Meanwhile, the interfacial crystal growth characterized by thin perovskite film was significantly affected by the ASMs (Figure 3f). Thin perovskite film on PTAA exhibited many pinholes. However, a few pinholes with a complete surface coverage of thin perovskite films formed after the introduction of ASMs due to the improved wettability of perovskite on ASMs. In this case, the improved interfacial crystal growth reduced the perovskite‐substrate defects and enhanced the hole transport properties between HTL and perovskite.

The impact of ASMs on the morphology of perovskite was explored by atomic force microscopy (AFM) in the scanning area 10 µm × 10 µm. As shown in Figure S2 (Supporting Information), the root‐mean‐square (RMS) values decreased from 11.1 to 8.9 nm after the growth of perovskite film on PTAA and DTAC, respectively. The resulting smooth surface could facilitate the contact between the perovskite and PCBM layer. Overall, the improved crystal growth and morphology of perovskite films based on ASMs contributed to declining the perovskite–substrate defects, suppressing charge recombination, and improving hole transport.

On the other hand, the UV–vis absorption spectra of ASMs showed no obvious influence on the absorption when compared to pure PTAA (Figure S3, Supporting Information). By contrast, the corresponding perovskite films deposited on ASMs displayed higher absorption at wavelengths of 350–750 nm when compared to the perovskite film deposited on PTAA, indicating the superior quality of perovskite film. This further confirmed the better crystal quality, conducive to improving the light‐harvesting ability for high external quantum efficiency (EQE) and short‐circuit current density (*J*_SC_).^[^ ^37^, ^38^ ^]^

**Figure 4:**
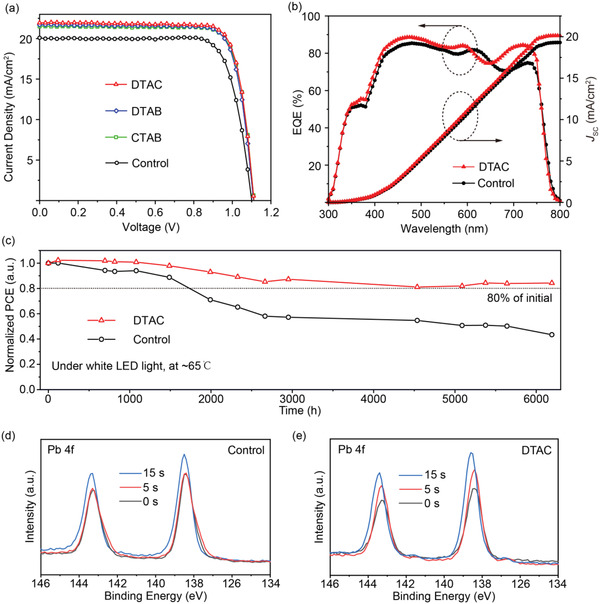
a) Current density–voltage (*J–V*) curves of optimized devices based on the control and ASMs. b) EQE and integrated *J*_SC_ of PSCs based on PTAA and DTAC. c) Photostabilities of devices with and without DTAC. d,e) XPS spectra of Pb element distribution as a function of etching time of argon ion (2 keV) obtained from the top surface of PTAA without and with DTAC to perovskite.

Based on the extraordinary effects of ASMs on the perovskite‐substrate, inverted PSCs consisting of ASMs were assembled and the current density–voltage (*J–V*) curves were gathered in **Figure** **4**a. The optimized PSC based on DTAC under reverse scanning delivered a PCE of 19.7% with *J*_SC_ of 21.95 mA cm^−2^, *V*_OC_ of 1.11 V, and FF of 0.81. The recorded PCE value was 14% higher than that of control PSC based on PTAA (17.3%). The EQE spectrum of DTAC‐based device was also consistent with that acquired from the *J–V* curve (Figure 4b). The DTAC‐derived PSC revealed no notable photocurrent hysteresis under forward and reverse scans (Figure S4 and Table S1, Supporting Information). The Hysteresis index (HI) ((PCE_reverse_ − PCE_forward_)/PCE_reverse_) of the device based on ASMs was calculated as 0.04, a value much lower than that of the control device (0.09).^[^ ^39^ ^]^ The reduced hysteresis behavior may be associated with the large grains and passivation of perovskite–substrate defects, thereby suppressing the ion migration (Figure 4d,e).^[^ ^40^ ^]^ Furthermore, the stabilized PCE and stabilized *J*_SC_ at a bias voltage of 0.93 V with maximum power point reached 19.0% and 20.44 mA cm^−2^, respectively (Figure S5, Supporting Information). The statistical measurements of photovoltaic parameters of 21 individual PSCs are summarized in Figure S6 and Table S2 (Supporting Information). The control devices showed a lower average PCE of 15.6% and a larger standard deviation of 0.91. By comparison, the devices based on ASMs showed better reproducibility and higher average PCE. The average PCE of PSCs based on DTAC was estimated to be 18.9% with a lower standard deviation of 0.35, demonstrating enhanced reproducibility, further verifying the improved PCE. The universality of such dynamic modulation strategy was explored by fabricating and testing DTAC‐derived MAPbI_3_ devices (Figure S7, Supporting Information). The maximum PCE of the DTAC‐based MAPbI_3_ device reached 18.5%, equivalent to a 9% improvement when compared to the control device (17.0%). Therefore, the proposed dynamic modulation process is promising as a universal method.

The long‐term photostability of the unencapsulated devices was further investigated under continuous 100 mW cm^−2^ irradiation of ≈65 °C without any ultraviolet filter. As shown in Figure 4c, the DTAC‐derived device retained ≈84% of the initial PCE under 1‐sun illumination for 6200 h, whereas the control devices decreased dramatically with only 43% left after the same period. The extrapolation revealed T_80_ reaching above 10 000 h for DTAC‐derived cells (Figure S8, Supporting Information). To the best of our knowledge, such photostability outperformed other previously reported inverted PSCs (Table S3, Supporting Information). Similarly, CTAB and DTAB‐derived devices retained ≈75% and 78% of the initial PCE under 1‐sun illumination for 6200 h, indicating our dynamic modulation strategy is an effective way for long‐term photostability (Figure S9, Supporting Information). Here, ion migration played a crucial role in photostability, and strong chemical interaction between ASMs and perovskite film could suppress the Pb ion migration for long‐term photostability.^[^ ^17^, ^41^ ^]^

The possible suppression of Pb ion migration by DTAC was experimentally studied by XPS measurements with argon ion etching to investigate the depth distribution of the Pb atomic from the top surface of PTAA/DTAC to the perovskite.^[^ ^42^, ^43^ ^]^ As shown in Figure 4d,e, the perovskite film based on PTAA showed strong Pb signal intensity that remained almost unchanged at different ion etching times. By comparison, the Pb signal intensity weakened after the introduction of DTAC, and the trend increased significantly as a function of argon ion etching time. Thus, the DTAC could effectively suppress the Pb ion migration. I ion signal also showed the same tendency as Pb ion (Figure S10, Supporting Information), demonstrating the suppressed I ion migration. To further prove the suppression of ion migration, ion migration activation energy (*E*_a_) was also measured (Figure S11, Supporting Information). The *E*_a_ of control and DTAC‐derived perovskite films is 0.45 and 0.57 eV, respectively. The higher *E*_a_ values also indicated that DTAC could suppress the ion migration. Note that the photostability tests were performed under white LED at temperatures reaching as high as 65 °C. Under these conditions, dynamic modulation could reduce the interface strain and defects (Figure 1b), one of the reasons for the improved photostability. These results also suggested that improving the photostability was not only linked to ions migration, but also to the interface strain that cannot be ignored.^[^ ^18^ ^]^ Meanwhile, the improved quality of perovskite film could enhance the photostability of PSCs.

**Figure 5:**
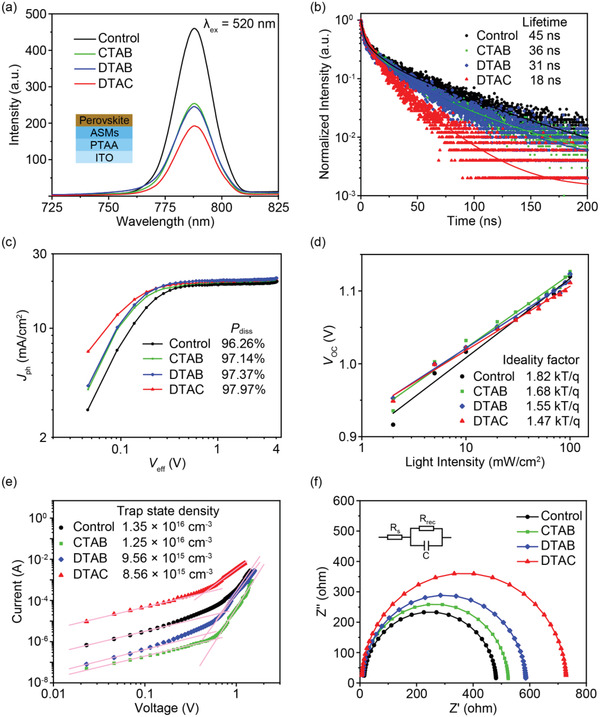
a) Steady‐state PL and b) PL decay profiles of perovskite film deposited on different surfaces. c) The change in photocurrent (*J*_ph_) as a function of applied voltage (*V*_eff_) for the corresponding PSCs to calculate the exciton dissociation probability (*P*_diss_). d) The dependence of *V*_OC_ on the light intensity curves. e) *J–V* curves of the hole‐only devices used to estimate the defect concentrations of perovskite films. f) Nyquist plots of corresponding PSCs under dark at 1.1 V.

The reasons for the improved performances were clarified by studying the photophysical and optoelectrical properties of perovskite deposited on different surfaces. The steady‐state photoluminescence (PL) spectra and PL decay profiles of perovskite films deposited on PTAA and ASMs are shown in **Figure** **5**a,b. The strongest PL quenching of perovskite based on DTAC with the shortest lifetime of 18 ns indicated the contribution of DTAC to improving the hole‐extraction and transportation ability from perovskite to HTL.^[^ ^44^ ^]^ The charge collection probability of HTL was studied by drawing the plots of photocurrent (*J*_ph_) at different effective applied voltages (*V*_eff_) for PSCs based on PTAA and dynamic modulation as displayed in Figure 5c. Here, *J*_ph_ was defined by the equation: *J*_ph_ = *J*_L_ − *J*_D_, where *J*_L_ and *J*_D_ are obtained by *J–V* curves under the illumination of AM1.5G and darkness, respectively. Also, *V*_eff_ was determined by the equation: *V*_eff_ = *V*_0_ − *V*_app_, where *V*_0_ and *V*_app_ represent the voltages at *J*_L_ equals to *J*_D_ and applied bias, respectively. The exciton dissociation probability (*P*_diss_) could be calculated by the following equation: *P*_diss_ = *J*_ph_/*J*_sat_, where *J*_sat_ refers to saturate current density.^[^ ^45^, ^46^ ^]^ The device with DTAC exhibited the highest *P*_diss_ of 97.97%, while the control device displayed the lowest *P*_diss_ of 96.26%. The excellent exciton dissociation probability of devices based on dynamic modulation indicated the contribution of ASMs to hole‐extraction and transportation, leading to reduced charge recombination.

The built‐in potential (*V*_bi_) was further investigated by Mott–Schottky plots for PSCs based on PTAA and DTAC, and data revealed that the introduction of the DTAC enhanced *V*_bi_ when compared to the control device (Figure S12, Supporting Information). This also demonstrated the increase in exciton dissociation probability for the PSCs after the introduction of ASMs.^[^ ^38^ ^]^ Moreover, the effects of trap‐assisted recombination on the devices were evaluated by investigating the dependence of *V*_OC_ on the light intensity. As shown in Figure 5d, the ASMs‐derived PSCs showed smaller ideality factors of 1.68, 1.55, and 1.47 for respectively CTAB, DTAB, and DTAC when compared to the control device (1.82). The reduced ideality factor indicated that ASMs could effectively suppress the trap‐assisted recombination due to interfacial defect passivation by ASMs.^[^ ^47^ ^]^

The trap state density was further identified by the space charge limit current (SCLC) analysis performed on a hole‐only device (Figure 5e). Note that the obtained dark *J–V* curves could be used to calculate the trap‐filled limited voltage (*V*_TFL_). Compared to control device (*V*_TFL_ = 0.68 V), the ASMs‐derived devices showed lower *V*_TFL_ values of 0.63, 0.48, and 0.43 V for CTAB, DTAB, and DTAC, respectively. The corresponding trap state densities for the control, CTAB, DTAB, and DTAC‐derived perovskite films were calculated as 1.35 × 10^16^, 1.25 × 10^16^, 9.56 × 10^15^, and 8.56 × 10^15^ cm^–3^, respectively. Electron‐only devices were further fabricated and measured (Figure S13, Supporting Information). The calculated trap state densities were 8.76 × 10^15^, 7.97 × 10^15^, 5.97 × 10^15^, and 4.18 × 10^15^ cm^–3^ for the control, CTAB, DTAB, and DTAC‐derived perovskite films, respectively. The reduced trap density should be attributed to the passivated perovskite–substrate defects and excellent crystal quality of perovskite films.^[^ ^34^ ^]^ Moreover, the charge recombination and transport processes at the interface between perovskite and HTL were explored by electrochemical impedance spectroscopy (EIS) measurements in Figure 5f. The detailed fitting EIS data are listed in Table S4 (Supporting Information). The junction capacitance and recombination resistance were determined by the middle frequency zone of EIS semicircle. The device based on DTAC showed the largest recombination resistance for suppressed charge recombination due to reduced interface defects, conductive to improved device performance.^[^ ^44^ ^]^ Meanwhile, Nyquist plots of corresponding PSCs under 1 sun illumination (AM 1.5G) were also measured (Figure S14, Supporting Information). From the plots, DTAC‐derived PSCs showed the smallest charge transport resistance (*R*_ct_) and arc, demonstrating good interfacial contact between perovskite and PTAA with the introduction of DTAC.

## 3 Conclusion

In summary, a novel dynamic modulation strategy was successfully developed to fabricate high‐quality perovskite films for highly stable inverted PSCs by constructing soft perovskite–substrate interfaces using amphiphilic soft long‐chain molecules. The ASMs were suitable for releasing residual strain at perovskite‐substrate interfaces due to the soft self‐adaptive long‐chains, as well as facilitating the nucleation and growth of perovskite crystals by improving the wettability of PTAA, passivating defects at perovskite–substrate interfaces by forming chemical interactions between the Lewis base group of amphiphilic molecules and undercoordinated Pb^2+^ of perovskite film. As a result, high‐quality perovskite films were obtained and the unencapsulated device showed state‐of‐the‐art photostability in inverted planner PSCs, maintaining over 84% of the initial efficiency under continuous 1‐sun illumination of ≈65 °C for 6200 h. Moreover, the resultant devices reached efficiencies approaching 20% with good reproducibility and negligible hysteresis behavior. These findings look very promising of soft perovskite–substrate interfaces in constructing high‐quality perovskite films with improved wettability, suppressed interfacial defects, and reduced residual strain for achieving highly stable and efficient perovskite optoelectronic devices for future commercialization.

## Conflict of Interest

The authors declare no conflict of interest.

## Supporting information

Supporting Information

## Data Availability

The data that support the findings of this study are available from the corresponding author upon reasonable request.

## References

[1] Q. Dong , Y. Fang , Y. Shao , P. Mulligan , J. Qiu , L. Cao , J. Huang , Science 2015, 347, 967. 25636799 10.1126/science.aaa5760

[2] J. H. Noh , S. H. Im , J. H. Heo , T. N. Mandal , S. I. Seok , Nano Lett. 2013, 13, 1764. 23517331 10.1021/nl400349b

[3] C. S. Ponseca , T. J. Savenije , M. Abdellah , K. Zheng , A. Yartsev , T. Pascher , T. Harlang , P. Chabera , T. Pullerits , A. Stepanov , J. Wolf , V. Sundström , J. Am. Chem. Soc. 2014, 136, 5189. 24654882 10.1021/ja412583t

[4] J. S. Manser , J. A. Christians , P. V. Kamat , Chem. Rev. 2016, 116, 12956. 27327168 10.1021/acs.chemrev.6b00136

[5] L. Xu , D. Wu , W. Lv , Y. Xiang , Y. Liu , Y. Tao , J. Yin , M. Qian , P. Li , L. Zhang , S. Chen , O. F. Mohammed , O. M. Bakr , Z. Duan , R. Chen , W. Huang , Adv. Mater. 2022, 34, 2107111. 10.1002/adma.202107111 34739745

[6] L. Xu , Y. Li , C. Zhang , Y. Liu , C. Zheng , W. Lv , M. Li , Y. Chen , W. Huang , R. Chen , Sol. Energy Mater. Sol. Cells 2020, 206, 110316.

[7] Z. Liu , L. Qiu , L. K. Ono , S. He , Z. Hu , M. Jiang , G. Tong , Z. Wu , Y. Jiang , D. Son , Y. Dang , S. Kazaoui , Y. Qi , Nat. Energy 2020, 5, 596.

[8] Y. Lin , N. Sakai , P. Da , J. Wu , H. C. Sansom , A. J. Ramadan , S. Mahesh , J. Liu , R. D. J. Oliver , J. Lim , L. Aspitarte , K. Sharma , P. K. Madhu , A. B. Morales Vilches , P. K. Nayak , S. Bai , F. Gao , C. R. M. Grovenor , M. B. Johnston , J. G. Labram , J. R. Durrant , J. M. Ball , B. Wenger , B. Stannowski , H. J. Snaith , Science 2020, 369, 96. 32631893 10.1126/science.aba1628

[9] Y. Lin , Y. Liu , S. Chen , S. Wang , Z. Ni , C. H. Van Brackle , S. Yang , J. Zhao , Z. Yu , X. Dai , Q. Wang , Y. Deng , J. Huang , Energy Environ. Sci. 2021, 14, 1563.

[10] Y. Zhao , T. Heumueller , J. Zhang , J. Luo , O. Kasian , S. Langner , C. Kupfer , B. Liu , Y. Zhong , J. Elia , A. Osvet , J. Wu , C. Liu , Z. Wan , C. Jia , N. Li , J. Hauch , C. J. Brabec , Nat. Energy 2022, 7, 144.

[11] Y. Zhao , P. Miao , J. Elia , H. Hu , X. Wang , T. Heumueller , Y. Hou , G. J. Matt , A. Osvet , Y. Chen , M. Tarragó , D. de Ligny , T. Przybilla , P. Denninger , J. Will , J. Zhang , X. Tang , N. Li , C. He , A. Pan , A. J. Meixner , E. Spiecker , D. Zhang , C. J. Brabec , Nat. Commun. 2020, 11, 6328. 33303755 10.1038/s41467-020-20066-7 PMC7730187

[12] T. Ishii , A. Masuda , Prog. Photovoltaics 2017, 25, 953.

[13] https://en.longi‐solar.com/home/products/technology.html, 2022.

[14] I. Hwang , I. Jeong , J. Lee , M. J. Ko , K. Yong , ACS Appl. Mater. Interfaces 2015, 7, 17330. 26154828 10.1021/acsami.5b04490

[15] S. Ma , Y. Bai , H. Wang , H. Zai , J. Wu , L. Li , S. Xiang , N. Liu , L. Liu , C. Zhu , G. Liu , X. Niu , H. Chen , H. Zhou , Y. Li , Q. Chen , Adv. Energy Mater. 2020, 10, 1902472.

[16] L. Shi , M. P. Bucknall , T. L. Young , M. Zhang , L. Hu , J. Bing , D. S. Lee , J. Kim , T. Wu , N. Takamure , D. R. Mckenzie , S. Huang , M. A. Green , A. W. Y. Ho‐Baillie , Science 2020, 368, eaba2412. 32439657 10.1126/science.aba2412

[17] H. Zai , Y. Ma , Q. Chen , H. Zhou , J. Energy Chem. 2021, 63, 528.

[18] Y. Cheng , L. Ding , Energy Environ. Sci. 2021, 14, 3233.

[19] Y. H. Deng , X. P. Zheng , Y. Bai , Q. Wang , J. J. Zhao , J. S. Huang , Nat. Energy 2018, 3, 560.

[20] L. Xu , C. Zhang , X. Feng , W. Lv , Z. Huang , W. Lv , C. Zheng , G. Xing , W. Huang , R. Chen , J. Mater. Chem. A 2021, 9, 16943.

[21] H. Zhang , M. Qin , Z. Chen , W. Yu , Z. Ren , K. Liu , J. Huang , Y. Zhang , Q. Liang , H. T. Chandran , P. W. K. Fong , Z. Zheng , X. Lu , G. Li , Adv. Mater. 2021, 33, 2100009. 10.1002/adma.202100009 33893688

[22] Q. Zhou , J. Qiu , Y. Wang , S. Li , M. Yu , J. Liu , X. Zhang , Chem. Eng. J. 2022, 440, 135974.

[23] N. Li , X. Niu , L. Li , H. Wang , Z. Huang , Y. Zhang , Y. Chen , X. Zhang , C. Zhu , H. Zai , Y. Bai , S. Ma , H. Liu , X. Liu , Z. Guo , G. Liu , R. Fan , H. Chen , J. Wang , Y. Lun , X. Wang , J. Hong , H. Xie , D. S. Jakob , X. G. Xu , Q. Chen , H. Zhou , Science 2021, 373, 561. 34326239 10.1126/science.abh3884

[24] E. D. Jung , A. K. Harit , D. H. Kim , C. H. Jang , J. H. Park , S. Cho , M. H. Song , H. Y. Woo , Adv. Mater. 2020, 32, 2002333. 10.1002/adma.202002333 32567159

[25] C. Bi , Q. Wang , Y. Shao , Y. Yuan , Z. Xiao , J. Huang , Nat. Commun. 2015, 6, 7747. 26190275 10.1038/ncomms8747 PMC4518278

[26] F. Li , X. Deng , F. Qi , Z. Li , D. Liu , D. Shen , M. Qin , S. Wu , F. Lin , S. Jang , J. Zhang , X. Lu , D. Lei , C. Lee , Z. Zhu , A. K. Y. Jen , J. Am. Chem. Soc. 2020, 142, 20134. 33190487 10.1021/jacs.0c09845

[27] B. Chen , P. N. Rudd , S. Yang , Y. Yuan , J. Huang , Chem. Soc. Rev. 2019, 48, 3842. 31187791 10.1039/c8cs00853a

[28] L. Fu , H. Li , L. Wang , R. Yin , B. Li , L. Yin , Energy Environ. Sci. 2020, 13, 4017.

[29] J. Wu , S. Liu , Z. Li , S. Wang , D. Xue , Y. Lin , J. Hu , Natl. Sci. Rev. 2021, 8, 047. 10.1093/nsr/nwab047 PMC8363326 34691711

[30] D. Liu , D. Luo , A. N. Iqbal , K. W. P. Orr , T. A. S. Doherty , Z. Lu , S. D. Stranks , W. Zhang , Nat. Mater. 2021, 20, 1337. 34531574 10.1038/s41563-021-01097-x

[31] L. Xu , M. Qian , C. Zhang , W. Lv , J. Jin , J. Zhang , C. Zheng , M. Li , R. Chen , W. Huang , Nano Energy 2020, 67, 104244.

[32] J. Fang , Z. Ding , X. Chang , J. Lu , T. Yang , J. Wen , Y. Fan , Y. Zhang , T. Luo , Y. Chen , S. F. Liu , K. Zhao , J. Mater. Chem. A 2021, 9, 13297.

[33] Y. Wang , T. Wu , J. Barbaud , W. Kong , D. Cui , H. Chen , X. Yang , L. Han , Science 2019, 365, 687. 31416961 10.1126/science.aax8018

[34] Y. Cai , J. Cui , M. Chen , M. Zhang , Y. Han , F. Qian , H. Zhao , S. Yang , Z. Yang , H. Bian , T. Wang , K. Guo , M. Cai , S. Dai , Z. Liu , S. F. Liu , Adv. Funct. Mater. 2021, 31, 2005776.

[35] Q. Yang , X. Wang , S. Yu , X. Liu , P. Gao , X. Hu , G. Hou , S. Chen , X. Guo , C. Li , Adv. Energy Mater. 2021, 11, 2100493.

[36] L. Jia , F. Huang , H. Ding , C. Niu , Y. Shang , W. Hu , X. Li , X. Yu , X. Jiang , R. Cao , J. Zhu , G. Wang , M. Chen , S. Yang , Nano Today 2021, 39, 101164.

[37] J. Lee , H. Kang , G. Kim , H. Back , J. Kim , S. Hong , B. Park , E. Lee , K. Lee , Adv. Mater. 2017, 29, 1606363. 10.1002/adma.201606363 28394417

[38] J. Yang , Q. Cao , Z. He , X. Pu , T. Li , B. Gao , X. Li , Nano Energy 2021, 82, 105731.

[39] G. Yin , J. Ma , H. Jiang , J. Li , D. Yang , F. Gao , J. Zeng , Z. Liu , S. F. Liu , ACS Appl. Mater. Interfaces 2017, 9, 10752. 28291331 10.1021/acsami.7b01063

[40] Y. Wang , X. Liu , Z. Zhou , P. Ru , H. Chen , X. Yang , L. Han , Adv. Mater. 2018, 31, 1803231. 10.1002/adma.201803231 30663134

[41] J. Wei , Q. Wang , J. Huo , F. Gao , Z. Gan , Q. Zhao , H. Li , Adv. Energy Mater. 2021, 11, 2002326.

[42] L. Xu , Y. Liu , W. Qiu , Y. Li , H. Wang , M. Li , L. Xian , C. Zheng , Y. Chen , R. Chen , J. Power Sources 2021, 506, 230120.

[43] W. C. Lin , A. Kovalsky , Y. C. Wang , L. L. Wang , S. Goldberg , W. L. Kao , C. Y. Wu , H. Y. Chang , J. J. Shyue , C. Burda , Phys. Chem. Chem. Phys. 2017, 19, 21407. 28758661 10.1039/c7cp03116e

[44] C. Liu , L. Zhang , Y. Li , X. Zhou , S. She , X. Wang , Y. Tian , A. K. Y. Jen , B. Xu , Adv. Funct. Mater. 2020, 30, 1908462.

[45] H. Lai , D. Lu , Z. Xu , N. Zheng , Z. Xie , Y. Liu , Adv. Mater. 2020, 32, 2001470. 10.1002/adma.202001470 32627858

[46] G. Xu , R. Xue , S. J. Stuard , H. Ade , C. Zhang , J. Yao , Y. Li , Y. Li , Adv. Mater. 2021, 33, 2006753. 10.1002/adma.202006753 33634532

[47] B. Li , Y. Xiang , K. D. G. I. Jayawardena , D. Luo , Z. Wang , X. Yang , J. F. Watts , S. Hinder , M. T. Sajjad , T. Webb , H. Luo , I. Marko , H. Li , S. A. J. Thomson , R. Zhu , G. Shao , S. J. Sweeney , S. R. P. Silva , W. Zhang , Nano Energy 2020, 78, 105249.

