# Supplementary material for: Constructing Soft Perovskite–Substrate Interfaces for Dynamic Modulation of Perovskite Film in Inverted Solar Cells with Over 6200 Hours Photostability
Source: Adv Sci (Weinh). 2022 Aug 17;9(28):2202028. doi: 10.1002/advs.202202028 (PMC9534936; doi:10.1002/advs.202202028)
Supplement: Supplementary file 1 — Supporting Information [file ADVS-9-2202028-s001.pdf]

ADVANCED SCIENCE
Open Access

Supporting Information

for *Adv. Sci.*, DOI 10.1002/advs.202202028

Constructing Soft Perovskite–Substrate Interfaces for Dynamic Modulation of Perovskite Film in Inverted Solar Cells with Over 6200 Hours Photostability

Wenxuan Lv, Zhaoying Hu, Wei Qiu, Dongdong Yan, Meicheng Li, Anyi Mei, Ligang Xu and Runfeng Chen**

Supporting Information

Constructing soft perovskite-substrate interfaces for dynamic modulation of perovskite film in inverted solar cells with over 6,200 hours photostability

Wenxuan Lv, Zhaoying Hu, Wei Qiu, Dongdong Yan, Meicheng Li, Anyi Mei, Ligang Xu, Runfeng Chen**

W. Lv, Z. Hu, D. Yan, Prof. L. Xu, and Prof. R. Chen
Key Laboratory for Organic Electronics and Information Displays (KLOEID) & Jiangsu Key Laboratory for Biosensors, Institute of Advanced Materials (IAM), Nanjing University of Posts & Telecommunications, 9 Wenyuan Road, Nanjing, China.
E-mail: iamlgxu@njupt.edu.cn; iamrfchen@njupt.edu.cn

Prof. M. Li
State Key Laboratory of Alternate Electrical Power System with Renewable Energy Sources, School of New Energy, North China Electric Power University, Beijing, China.

Prof. A. Mei
Wuhan National Laboratory for Optoelectronics, Huazhong University of Science and Technology, Wuhan, Hubei, China.

1. Materials

N,N-Dimethylformamide (DMF, 99.5%) and dimethyl sulfoxide (DMSO, > 99.9%) were purchased from Alfa Aesar. Ethyl acetate (EA, > 98%) was from Acros Organics. Isopropanol (IPA) and toluene (Tol) were purchased from Energy Chemical Corp. Chlorobenzene (CB) was obtained from Aldrich [6,6]−Phenyl−C_{61}−butyric acid methyl ester (PCBM) and fullerene (C_{60}) were purchased from Luminescence Technology Corporation. LiF was obtained from Shanghai Han Feng Chemical, Co., LtD. Poly(triarylamines) (PTAA), CsI, $HC(NH_2)_2I$, CH_3NH_3Br, $PbBr_2$ and PbI_2 were from Xi'an Polymer Light Technology Co., LtD. Cetyltrimethylammonium bromide (CTAB), dodecyltrimethylammonium bromide (DTAB), and dodecyltrimethylammonium chloride (DTAC) were purchased from J&K Scientific. All these materials were used as received without purification.

2. Device fabrication

Indium tin oxide (ITO) coated glass was cleaned in deionized water, acetone, and ethanol subsequently and respectively for 10 min in an ultrasonicator (Shumei KQ300DE), then was treated with UV-ozone (ODT UV-O3 Cleaner) for 15 min. The PTAA was dissolved in toluene with a concentration of 4 mg mL^{-1} and spin-coated on the ITO substrate at 4000 rpm for 30 s, and then annealed on a hotplate at 100°C for 10 min. For the amphiphilic soft molecules (ASMs), CTAB, DTAB, and DTAC were dissolved in DMF (0.5 to 5 mg/mL) and spin-coated on PTAA at 6000 rpm for 70 s.

The perovskite precursor was dissolved in a mixed solvent (DMF/DMSO= 4:1 v/v) with a chemical formula of $(Cs_{0.05}FA_{0.81}MA_{0.14})Pb(I_{0.86}Br_{0.14})_3$. Then the precursor was spin-coated on the PTAA or ASMs at 10000 rpm for 55 s. During the spin-coating at the 40th s, 150 μL ethyl acetate was quickly dropped on the center of the substrates. The wet perovskite films were annealed at 100°C for 10 min. For the control device, DMF solution was spin-coated to treat PTAA before perovskite precursor spin-coating. Then, PCBM dissolved in chlorobenzene (20 mg/mL) was spin-coated at 2000 rpm for 60 s on the top surface of

perovskite layer. Finally, the C_{60} (~10 nm), LiF (~1 nm), and Cu (~100 nm) layers were thermally deposited in a vacuum chamber (< 5×10^{-4} Pa). C_{60} was deposited at a rate of 0.5 Å s^{-1} and the deposition rates of LiF and Cu were 0.1 and 10 Å s^{-1}, respectively.

3. Perovskite solar cell characterization

The current density and voltage (*J–V*) curves were obtained by a source meter (Keithley 2400) under AM1.5 sunlight at 100 mW cm^{-2} using a solar simulator (SAN-EI, XES-50S1). The National Renewable Energy Laboratories (NREL)-calibrated KG5 filtered silicon reference cell was applied to calibrate the AM1.5 irradiance level. The *J−V* curves were measured along the reverse scan direction from 1.2 V to −0.3 V and the forward scan direction from −0.3 V to 1.2 V at a scan rate of 100 mV·s^{-1}. All of the devices without encapsulation were tested in ambient air (25°C, ~40% relative humidity). During the *J-V* measurement, all PSCs were masked with a 0.08 cm^2 metal aperture. External quantum efficiency (EQE) measurements were carried out using custom-built Fourier transform photocurrent spectroscopy based on a QE-R system (Enli Tech.). The *J−V* curves for exciton dissociation probability were measured along with the forward scan from -3 to 1.5 V by a source meter (Keithley 2400) under the illumination of AM1.5G and in the dark.

The electrochemical impedance spectra (EIS) were measured on a CHI604 electrochemical work station (CH Instrument Inc.). A 5 mV voltage amplitude was applied at an applied bias voltage of 1.1 V with frequencies between 10^5 and 100 Hz under dark conditions. Meanwhile, EIS of the corresponding devices were further measured under 1 sun illumination (AM 1.5G) with frequencies between 106 and 100 Hz. The results were fitted using the software of Zsim. All characterizations and measurements were performed in ambient conditions. Mott–Schottky fitted capacitance-voltage plots were measured on a CHI604 electrochemical work station (CH Instrument Inc.). A 10 mV voltage amplitude was applied at different direct current voltages ranging from -0.1 to 1.2 V with a frequency of 10^3 Hz under dark conditions. The V_{bi} could be obtained from the C–V plots on the basis of Mott–

Schottky relationship $C^{-2} = ((V_{bi} - V)/A^2 e\varepsilon\varepsilon_0 N)$, where C is the depletion-layer capacitance, A is the active area, e is the elementary charge, ε and ε_0 represent the relative dielectric constant of the perovskite and vacuum dielectric constant, N is the doping density of perovskite, and V is the applied external voltage.

4. Device stability tests

The unencapsulated and unsealed devices were illuminated under continuous 100 mW cm^{-2} irradiation by a LED grow light without any ultraviolet filter (Spectrum King MLH140) monitored at open-circuit condition (without maximum power point tracking) at ~65°C in N_2. During the 6,200 h, the *J-V* curves were investigated at a time (for example 20 h or several days) for investigating the stability. The National Renewable Energy Laboratories (NREL)-calibrated KG5 filtered silicon reference cell was applied to calibrate the AM1.5 irradiance level.

5. Perovskite film characterization

The morphologies of the samples were characterized by scanning electron microscopy (SEM, Hitachi S-4800) and atomic force microscope (AFM). AFM was carried out using an FM-Nanoview 1000 equipped with Scanasyst-Air peak force tapping mode AFM tips from FSM-Precision Co., Ltd. The chemical compositions and structures of the perovskite films were analyzed by X-ray diffraction (Rigaku Smartlab X). X-ray photoelectron spectra (XPS) analysis was performed on an ESCALAB 250 system equipped with a monochromatic Al Kα X-ray source ($h\nu$=1486.6 eV). For XPS etching measurements, DTAC was dissolved in IPA to avoid dissolving the perovskite film. The steady-state photoluminescence (PL) spectra and time-resolved PL decays of the perovskite films on ITO/PTAA substrates were measured by an Edinburgh FLS 980. A 450 nm pulse laser with a repetition frequency of ~1–20 MHz was employed to measure the PL lifetime by an equation, $I=I_0\times\exp[-(t/\tau)^{\beta}]$, where I and I_0 are the PL intensity and the initial intensity at time zero, τ is the lifetime and β is the stretching exponent ($0 < \beta < 1$). UV-vis absorption spectra were obtained on a Jasco V-750 UV-

Visible/Near-Infrared Spectrophotometers. Contact angle characterizations were carried out on PTAA and ASMs using droplet shape analyzer (KRÜSS GmbH Germany). X-ray diffraction (XRD) of perovskite films were conducted on Rigaku Smartlab X-Ray diffractometer. 2θ were measured from 10° to 40° with an increment of 0.02°/0.1 s. In Williamson-Hall analysis, the strain (ε) is derived from the following equation: $\beta\cos\theta = \frac{k\lambda}{D} + 4\varepsilon sin\theta$, where β is the crystallite size and can be calculated from the full width at half maximum (FWHM) of the peaks (100) (110) (111) (200) (210), θ is Bragg diffraction angle, k is Scherrer constant, λ is X-ray wavelength, and D is the average thickness of the crystal grain.

The hole-only and electron-only devices were fabricated with the structure of ITO/HTM/Perovskite/MoO_3/Cu and ITO/SnO_2/ASM/Perovskite/PCBM/Cu, respectively, to investigate the passivating properties of the ASMs. *J-V* curves were measured from 0 to 7 V with a step size of 0.02 V under dark conditions using the *J-V* sweep mode on a Keithley 2400 source/meter unit. From the *J-V* curves, trap state density (n_{trap}) can be calculated by the following equation according to the space-charge-limited current model:

$$n_{trap} = \frac{2\varepsilon\varepsilon_0 V_{TFL}}{qL^2} \quad (1)$$

where ε_0 is the vacuum dielectric constant, ε is the relative dielectric constant of perovskite, V_{TFL} is the trap filling limited (TFL) voltage, L is the film thickness.

The ion migration activation energy was extracted from the dependence of the conductivity of the perovskite films on temperature. In short, we used a lateral structure device which consists of two Cu electrodes with a length 12 mm and spacing gap of 1 mm deposited on surface of perovskite films with and without DTAC layer. In measurement, a constant bias of 0.5 V was applied for all the devices. The measurements were performed in a Lakeshore Probe Station. The samples were placed on a metal plate with the controllable temperature by

a heater. For each conductivity, the current through the devices were stabilized for 5 minutes when an objective temperature was reached, before the current measurement was performed. A semiconductor analyzer (Keithley 2400) was used for the current measurement with the applied bias of 0.5 V

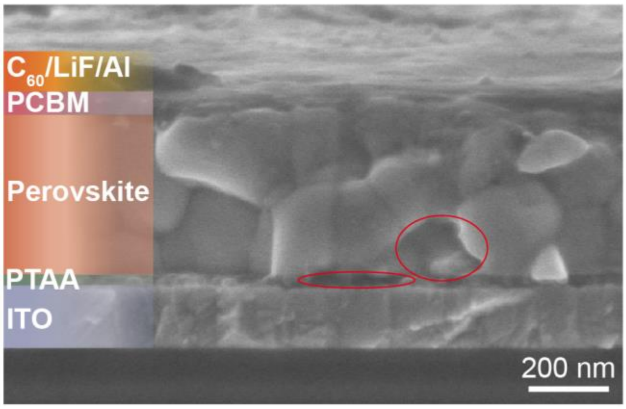

Figure S1 Cross-sectional SEM image of control inverted device based on PTAA

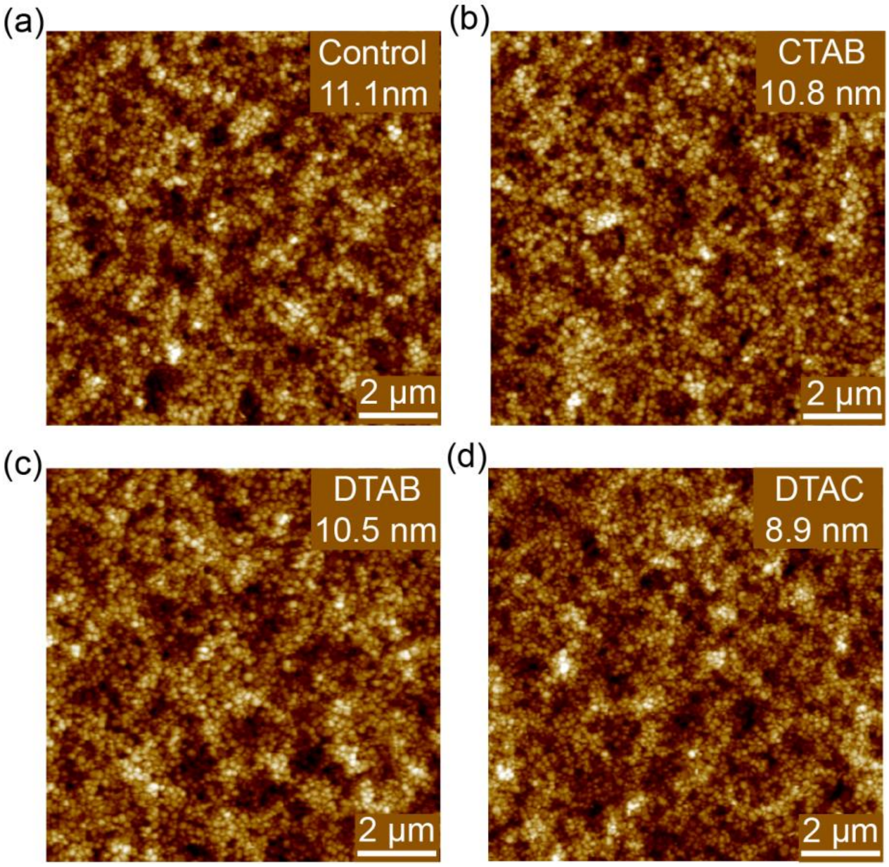

Figure S2 AFM images of perovskite films on (a) PTAA, (b) CTAB, (c) DTAB, (d) DTAC.

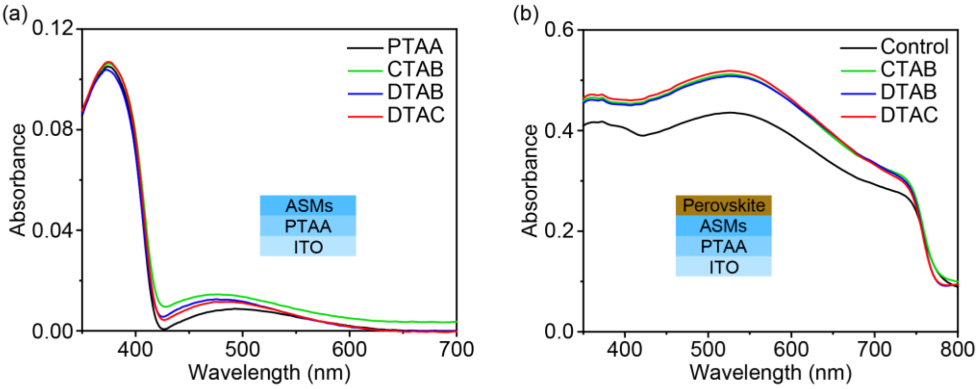

Figure S3 UV-vis absorption spectra of (a) PTAA or ASMs and (b) perovskite films on different ASMs.

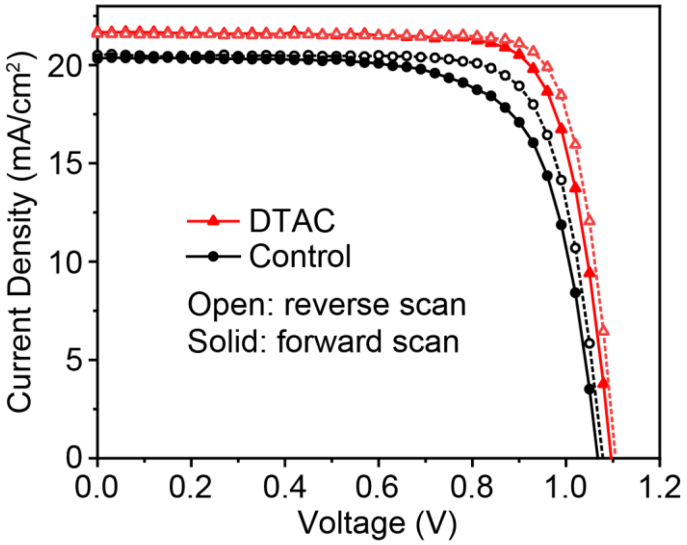

Figure S4 *J–V* curves measured in both forward and reverse scans.

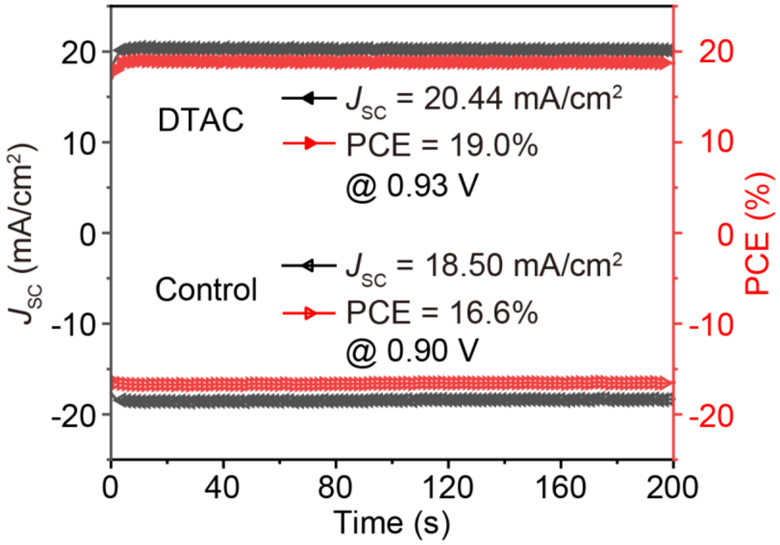

Figure S5 Stabilized PCE of the control and optimized device at the maximum power point of 0.90 and 0.93 V, respectively.

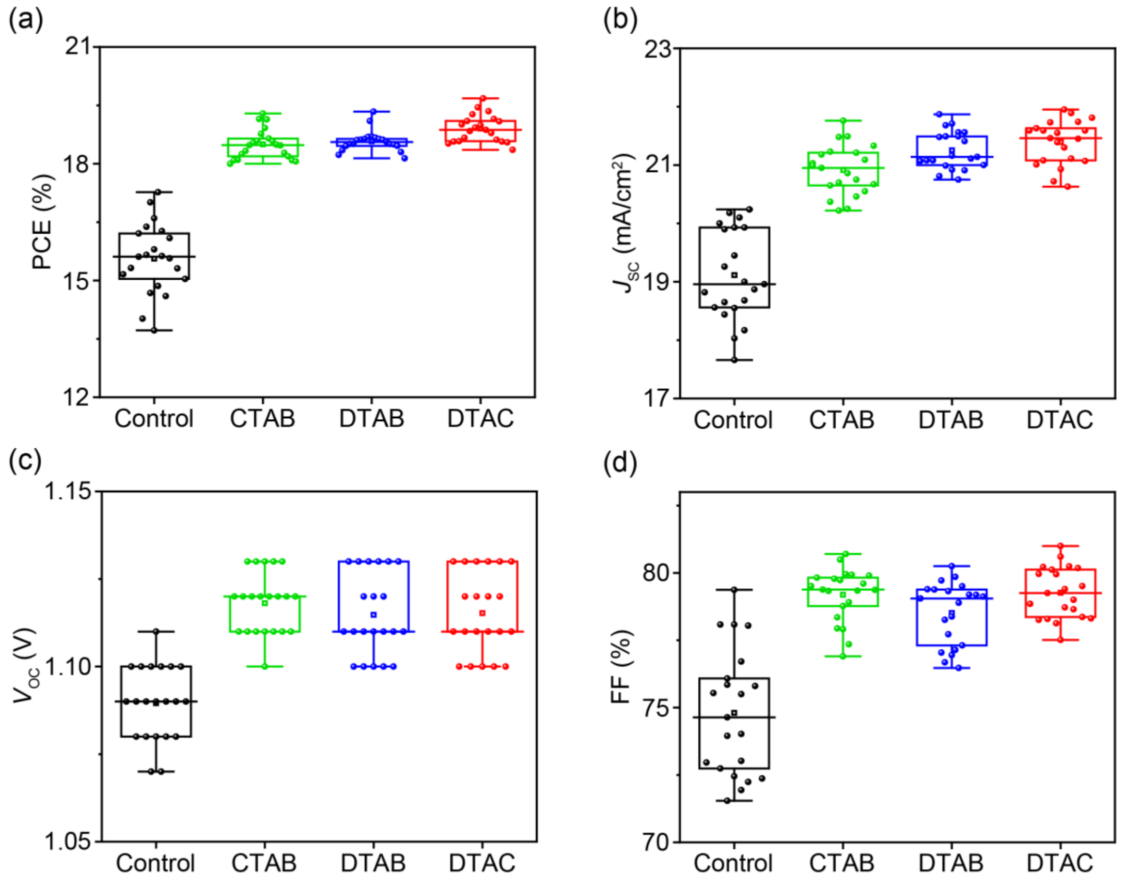

Figure S6 Histograms of (a) PCE, (b) J_{SC}, (c) V_{OC}, and (d) FF for PSCs without/with ASMs (based on 21 separate measurements).

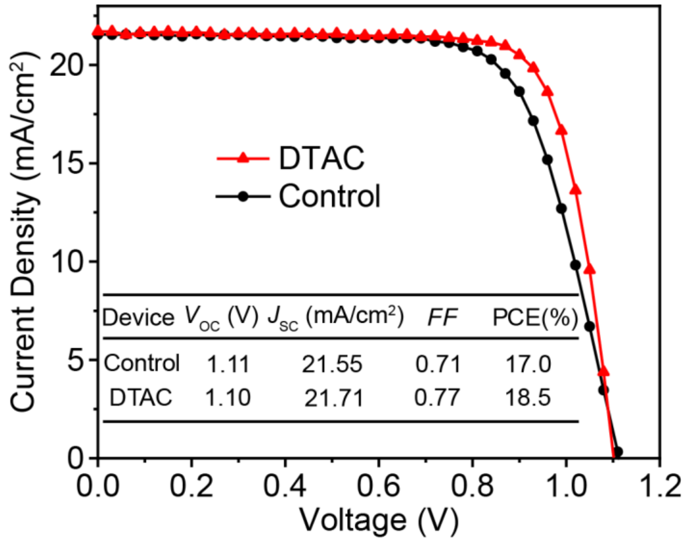

Figure S7 J–V curves of $MAPbI_3$-based PSCs based on control and DTAC.

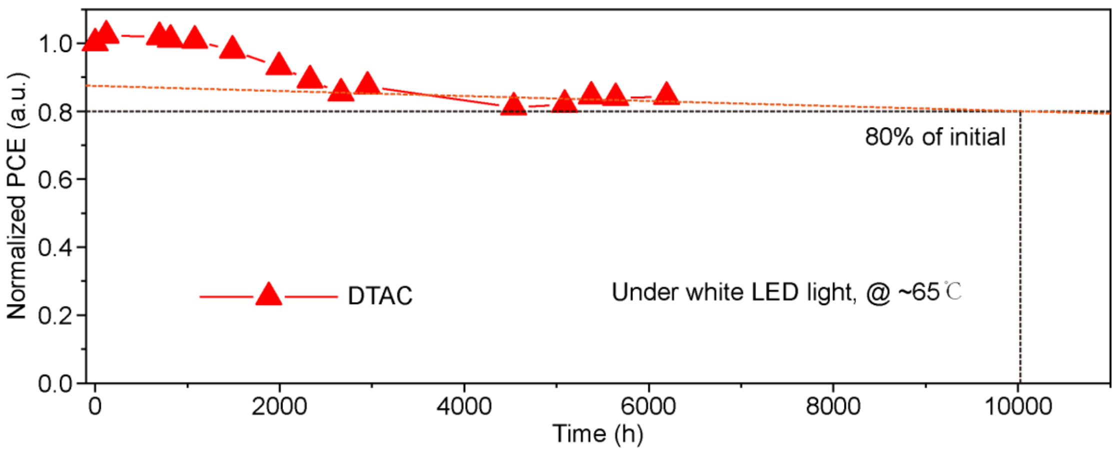

Figure S8 Estimated stability of device with DTAC.

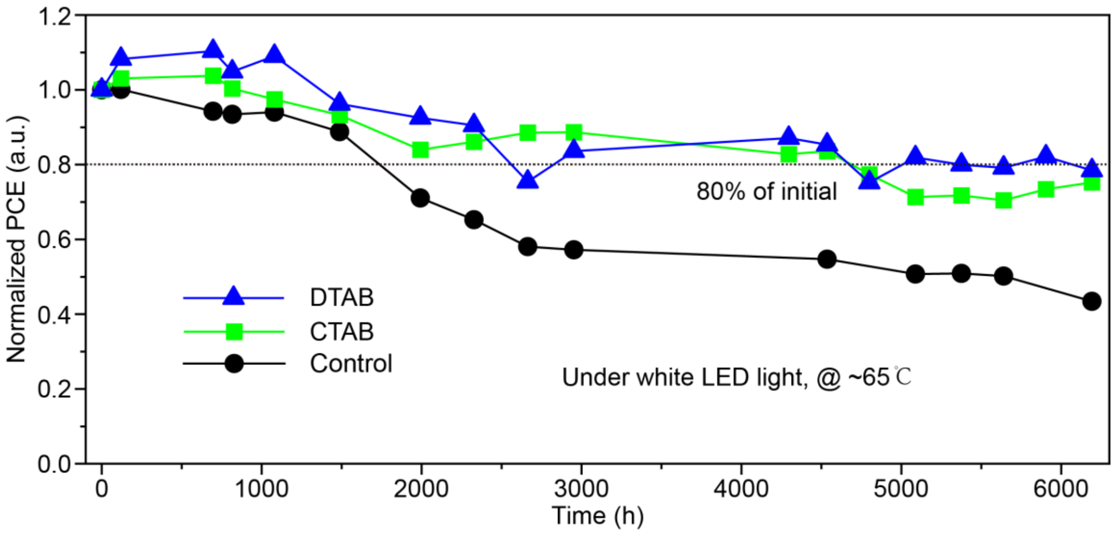

Figure S9 Photostability of the devices based on CTAB and DTAB.

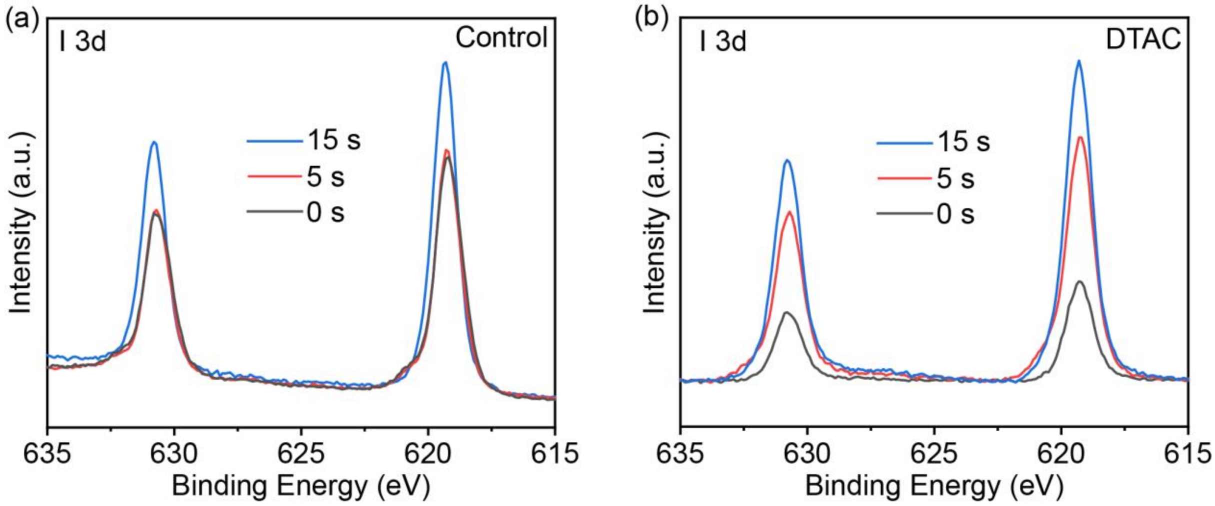

Figure S10 XPS spectra of I element distribution as a function of etching time of argon ion (2 KeV) obtained from the top surface of PTAA without and with DTAC to perovskite.

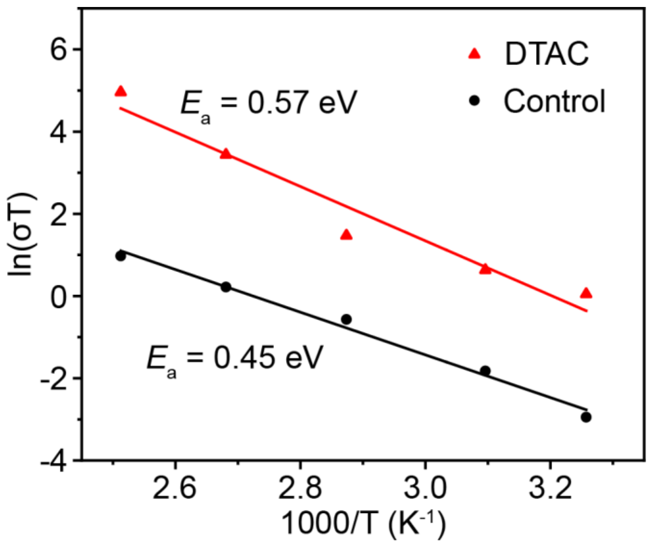

Figure S11 Ion migration activation energy (E_a) of control and DTAC-derived perovskite.

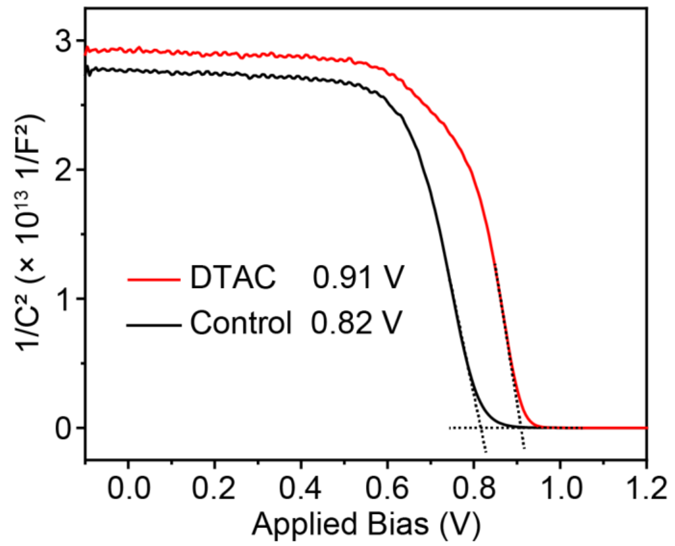

Figure S12 Mott–Schottky fitted capacitance-voltage plots of PSCs based on control and DTAC.

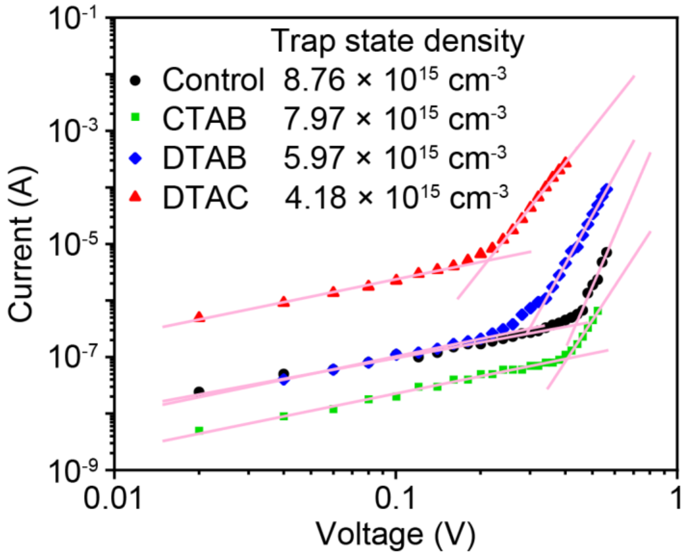

Figure S13 *J*–*V* curves of the electron-only devices used to estimate the defect concentrations of perovskite films.

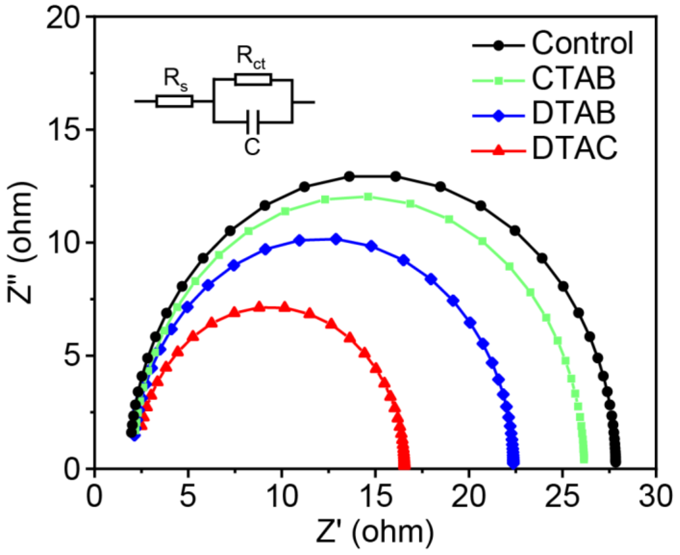

Figure S14. Nyquist plots of corresponding PSCs under 1 sun illumination (AM 1.5G). Inset shows the equivalent circuit diagram.

Table S1 Photovoltaic parameters of PSCs from forward and reverse scan with/without DTAC.

Sample	Scan direction	V_{OC} [V]	J_{SC} [mA cm^{-2}]	*FF*	PCE [%]	HI
Control	Forward	1.07	20.33	0.71	15.5	0.09
	Reverse	1.08	20.51	0.77	17.1	
DTAC	Forward	1.10	21.69	0.78	18.5	0.04
	Reverse	1.11	21.59	0.82	19.2	

Table S2. Summary of photovoltaic parameters of PSCs without and with ASMs.

Sample	V_{oc} [V]	J_{sc} [mA/cm^2]	*FF*	PCE [%]
Control	1.09±0.01 (1.10)	19.11±0.77 (20.10)	0.75±2.35 (0.78)	15.6±0.91 (17.3)
CTAB	1.12±0.01 (1.11)	20.92±0.43 (21.49)	0.79±1.00 (0.81)	18.5±0.38 (19.3)
DTAB	1.11±0.01 (1.11)	21.25±0.32 (21.71)	0.79±1.17 (0.80)	18.6±0.26 (19.3)
DTAC	1.12±0.01 (1.11)	21.39±0.38 (21.95)	0.79±0.95 (0.81)	18.9±0.35 (19.7)

Table S3. Comparison of the photostability of the inverted PSCs with recent reports. PCE_0 and PCE_t are the efficiencies before and after the stability test.

Device structure	Aging condition	Encapsulation	Testing duration	PCE_t/ PCE_0	Publishing date	Ref
ITO/PTAA/Perovskite/C_{60}/ BCP/Ag	white LED, 100 mW cm^{-2}, MPP, in N_2, 40-55℃	No	3,072 h	95.3%	Sep. 2021	[1]
ITO/PTAA/Perovskite/C_{60}/ BCP/Cu	continuous illumination at 1 sun intensity, with ultra-violet, in air, 65℃	Yes	2,180 h	93%	Jan. 2021	[2]
ITO/P3HT-COOH/Perovskite/5F-PCBP/PEI/Ag	constant 1 sun illumination with an AM1.5G filter, in N_2	No	1,920 h	80%	Aug. 2021	[3]
ITO/HTL/Perovskite/PCBM /BCP/Ag	continuous one-sun light illumination, in N_2	No	1,300 h	85%	Mar. 2021	[4]
FTO/PolyTPD:F4-TCNQ/Perovskite/PCBM/BCP/Cr/Au	xenon lamp, in air (60°C, RH~50±5%)	No	1,010 h	80%	Jul. 2020	[5]
	xenon lamp, in air (85°C, RH~50±5%)	Yes	1,200 h	95%		
ITO/NiO_x/Perovskite/PCBM/BCP/Cr/Au	xenon lamp, 70-75℃	Yes	1,200 h	92%	Oct. 2020	[6]
ITO/PTAA/Perovskite/PCBM/ZnO/Ag	continuous simulated AM 1.5G illumination, in N_2	Yes	1,000 h	91%	Oct. 2020	[7]
ITO/PTAA/Perovskite/C_{60}/ BCP/Cu	a 300 W plasma lamp with an adjusted light intensity of 100 mW cm^{-2}, room temperature	Yes	1,000 h	93%	May 2021	[8]
ITO/PTAA/Perovskite/PCBM/C_{60}/Cu	white LED, in N_2, ~65℃	No	6,200 h	84%	Apr. 2022	This work

Table S4. Fitting parameters of EIS under dark condition for the PSCs based on PTAA and ASMs.

Sample	R_s ($\Omega\cdot cm^2$)	C (F/cm^2)	R_{rec} ($\Omega\cdot cm^2$)
Control	14.0	6.65×10^{-8}	467.4
CTAB	9.4	5.97×10^{-8}	518.9
DTAB	8.6	6.20×10^{-8}	577.7
DTAC	6.2	6.32×10^{-8}	723.0

Reference

[1] Y. Zhan, F. Yang, W. Chen, H. Chen, Y. Shen, Y. Li, Y. Li, *Adv. Mater.* **2021**, *33*, 2105170.

[2] Y. Lin, Y. Liu, S. Chen, S. Wang, Z. Ni, C. H. Van Brackle, S. Yang, J. Zhao, Z. Yu, X. Dai, Q. Wang, Y. Deng, J. Huang, *Energy Environ. Sci.* **2021**, *14*, 1563.

[3] H. Huang, H. Tsai, R. Raja, S. Lin, D. Ghosh, C. Hou, J. Shyue, S. Tretiak, W. Chen, K. Lin, W. Nie, L. Wang, *ACS Energy Letters* **2021**, *6*, 3376.

[4] Q. Yang, X. Wang, S. Yu, X. Liu, P. Gao, X. Hu, G. Hou, S. Chen, X. Guo, C. Li, *Adv. Energy Mater.* **2021**, *11*, 2100493.

[5] Y. Lin, N. Sakai, P. Da, J. Wu, H. C. Sansom, A. J. Ramadan, S. Mahesh, J. Liu, R. D. J. Oliver, J. Lim, L. Aspitarte, K. Sharma, P. K. Madhu, A. B. Morales Vilches, P. K. Nayak, S. Bai, F. Gao, C. R. M. Grovenor, M. B. Johnston, J. G. Labram, J. R. Durrant, J. M. Ball, B. Wenger, B. Stannowski, H. J. Snaith, *Science* **2020**, *369*, 96.

[6] S. Wang, B. Yang, J. Han, Z. He, T. Li, Q. Cao, J. Yang, J. Suo, X. Li, Z. Liu, S. F. Liu, C. Tang, A. Hagfeldt, *Energy Environ. Sci.* **2020**, *13*, 5068.

[7] D. Koo, Y. Cho, U. Kim, G. Jeong, J. Lee, J. Seo, C. Yang, H. Park, *Adv. Energy Mater.* **2020**, *10*, 2001920.

[8] Y. Lu, J. Zhong, Y. Yu, X. Chen, C. Yao, C. Zhang, M. Yang, W. Feng, Y. Jiang, Y. Tan, L. Gong, X. Wei, Y. Zhou, L. Wang, W. Wu, *Energy Environ. Sci.* **2021**, *14*, 4048.
